# Towards developing a secure medical image sharing system based on zero trust principles and blockchain technology

**DOI:** 10.1186/s12911-020-01275-y

**Published:** 2020-10-07

**Authors:** Maliha Sultana, Afrida Hossain, Fabiha Laila, Kazi Abu Taher, Muhammad Nazrul Islam

**Affiliations:** 1 Department of Computer Science Engineering, Military Institute of Science and Technology, Mirpur Cantonment, Dhaka, 1216 Bangladesh; 2 Department of Information and Communication Technology, Bangladesh University of Professionals, Mirpur Cantonment, Dhaka, 1216 Bangladesh

**Keywords:** Medical Records, Medical Images, Electronic Health System, Blockchain, Zero Trust, Security, Inter Planetary File System

## Abstract

**Background:**

Data security has been a critical topic of research and discussion since the onset of data sharing in e-health systems. Although digitalization of data has increased efficiency and speed, it has also made data vulnerable to cyber attacks. Medical records in particular seem to be the regular victims of hackers. Several data breach incidents throughout history have warranted the invention of security measures against these threats. Although various security procedures like firewalls, virtual private networks, encryption, etc are present, a mix of these approaches are required for maximum security in medical image and data sharing.

**Methods:**

Relatively new, blockchain has become an effective tool for safeguarding sensitive information. However, to ensure overall protection of medical data (images), security measures have to be taken at each step, from the beginning, during and even after transmission of medical images which is ensured by zero trust security model. In this research, a number of studies that deal with these two concepts were studied and a decentralized and trustless framework was proposed by combining these two concepts for secured medical data and image transfer and storage.

**Results:**

Research output suggested blockchain technology ensures data integrity by maintaining an audit trail of every transaction while zero trust principles make sure the medical data is encrypted and only authenticated users and devices interact with the network. Thus the proposed model solves a lot of vulnerabilities related to data security.

**Conclusions:**

A system to combat medical/health data vulnerabilities has been proposed. The system makes use of the immutability of blockchain, the additional security of zero trust principles, and the scalability of off chain data storage using Inter Planetary File Systems (IPFS). The adoption of this system suggests to enhance the security of medical or health data transmission.

## Background

Nowadays, about 7.7 billion people use the Internet on a daily basis [1]. Its uses have transcended previous boundaries and veered into fields ranging from minute things like getting food delivered from one point to another to crucial functions like keeping track of money and online banking. With the increase of users, the congenial atmosphere of the Internet has morphed into one of malice [2–4].

The threats Internet users have been facing since the first data breach have remained the same. In this vein, Jung et al.[5] classified internet security into interception, fabrication, modification, and interruption. More specifically, they can be divided into two groups: unintentional errors (natural and man-made disasters and errors by employees) and intentional acts (fraud, identity theft, embezzlement, etc). Multiple instances of data breaches on the Internet [6, 7] have warranted discussions on the things that need to be considered when it comes to Internet security. With data breaches on the rise [8, 9], the need for an infallible solution was apparent. That solution came in the form of blockchain that provides protection against unwanted data exposure [10]. It is a distributed consensus mechanism that stores transaction information in a Peer-to-Peer (P2P) network [11]. Blockchain ensures digital trust by recording transactions in a public platform, while also making it immutable. Thus, it provides transparency, auditing and is also decentralized [12]. Currently blockchain is being implemented in various fields for data security, however, its use in the health sector to secure patient records is the most beneficial and crucial [13–15]. This is because health records are some of the most sensitive information out there and continues to be the victim of cyber attacks constantly [6]. Medical imaging devices in particular are the latest target of hackers [16] due to the lack of proper security measures taken around them [17]. Recent experiments by Israeli researchers show how easily MRI and CT scans can be tampered without any trace [18].

Advancements in cloud storage and cloud services has led to an increase in the mobile workforce and also provided an alternative to paper storage. Thus, security measures have to be taken at each step, from the beginning, during and even after transmission of data [19].

The zero trust security model addresses the aforementioned issues regarding security during every phase of data transmission. It is an IT security model that involves strict verification for users and devices trying to access resources on a network, regardless of whether they are sitting within or outside of the network perimeter [20]. No single specific technology is associated with zero trust, it is a holistic approach to network security that incorporates several different principles and technologies. Theoretically, blockchain is impenetrable, but it has its weaknesses. Blockchain cannot ensure protection against errors like: social engineering (an attack that involves the manipulation of people into ignoring security procedures and providing access to their data [21]), identity theft (stealing someone’s private key and accessing their accounts) [22], using weak passwords, and not patching known security vulnerabilities. Thus, it is important to enhance blockchain security by taking some extra measures such as: micro-segmentation, automated patch management, native data-at-rest encryption, and monitoring for changes to an application’s intended state and behaviour [23]. The traditional Moat-and-Castle security model for digital information can be enhanced using zero trust, which when implemented in a blockchain model would improve the overall security because blockchain will ensure transaction security, and the zero trust principles will improve access management, and user authentication [24].

Thus, the objective of this paper is to propose a decentralised, trustless and scalable framework which integrates the concepts of zero trust principles and blockchain. The proposed framework will facilitate to tackle data security issues and thus provide a safe way to transfer and store sensitive medical/health records and images.

A number of research has been conducted on the application of blockchain and zero trust models in various fields for data security.

The first ever functioning prototype has been proposed by Azaria et al.[25] which integrates blockchain to handle EMRs (Electronic Medical Record). Patients are given full control over their data. Medical stakeholders act as miners who are incentivized in two ways. Permissions associated with medical records are handled by smart contracts deployed on Ethereum. Later, data exchange is handled off chain between pre-existing centralized trusted databases. At every node, patient medical records are stored locally. In another study, Al Omar et al.[26] put forward a model which provides accountability, integrity, pseudonymity, security and privacy by storing encrypted medical data on blockchain and giving patients full control over their data. Here the data senders are the patients themselves and the data receivers are doctors, hospitals etc. Only registered users can communicate with the blockchain. It uses cryptography with blockchain to tackle data preserving vulnerabilities. Similarly, Dubovitskaya et al.[27] propose a framework that handles EMR data of cancer patients using permissioned blockchain. Data is stored off chain in a cloud-based storage. The nodes use PBFT (Practical Byzantine Fault Tolerance) consensus mechanism to validate each block. In another study, Dwivedi et al. [28] propose a model that integrates blockchain with IoT based wearable medical devices to share patient data. It uses a decentralized overlay network with several clusters instead of a single chain of blocks. The encrypted data is stored off chain in cloud storage. For preserving anonymity, ring signature is used. A study done by Vishal Patel includes blockchain to create a decentralized and secure system for sharing medical images. The medical images are kept at imaging centers and blockchain is used to regulate data viewing and sharing privileges [29].

The model put forward by Dey et al. [30] stands out because although they store data of patients measured by bio-sensors in blockchain, IPFS (Interplanetary File System) is used to save data of discharged patients to reduce the load on blockchain. It proposes an alternative solution to the traditional IoT model by using blockchain and encrypting communication between IoT devices.

The characteristics and the key concerns of implementation of a zero trust network are briefly discussed in [20] where Gilman and Barth stated that authentication and encryption of network flows, and at endpoints, enumeration of network flows, strength of the authentication and encryption techniques, public vs. private key infrastructures, and regular scanning and examining of device security are the aspects that need to be decided in the implementation of a zero trust network.

On the other hand, blockchain is considered a trust less system; but it does take some factors like device security, and intent of the miners for granted. Removing these elements of trust can fall under the domain of zero trust architecture. A very limited number of research has been conducted that has considered the use of blockchain and zero trust principles together. In [24], a model has been proposed using blockchain as an enabler in implementing the zero trust framework. The main focus of this framework was the implementation of zero trust architecture, using blockchain to ensure access management, user authentication and transaction security. In another work, Samaniego and Deters [31] proposed a model named Amatista, incorporating zero trust in blockchain for using it as a middleware for IoT devices. The zero trust hierarchical mining process was used in this model which puts block and transaction validation at different levels of trust.

In sum, the literature reviews suggest three important things. Firstly, it is seen that although blockchain has been used for maintaining health records, most rely on storing the data either directly on blockchain which is not scalable or on off chain storage systems that are not fully decentralised or compatible with blockchain. Moreover very few works have been done regarding large files like medical imaging data. Secondly, because zero trust architecture is relatively new, not much work has been done with it although implementation of its concepts will result in a more secured model. And finally, incorporation of the two concepts have lightly been talked about. The only model that combines blockchain with zero trust principles, does not fully explore the potential of enhancing security. Thus this paper focuses on developing a framework which is scalable, trustless and fully decentralised to ensure a secured process to transfer and store medical data by integrating blockchain and zero trust principles.

## Methods

To attain the research objective, a conceptual model is proposed by integrating the concepts of zero trust principles and blockchain. The proposed model deals with two users that share any medical/health data. In case of medical image sharing, medical technologist (person responsible for generating X-ray files, MRI scans etc.) acts as the sender, patient as the receiver and the data in question will be medical image files. The patient can also share data with a doctor in which case patient becomes the sender and doctor the receiver.

The following subsections discuss how zero trust principles and blockchain are individually implemented in the model and later an overview of the integrated system as a whole is provided.

### Zero trust principles

**Fig. 1:**
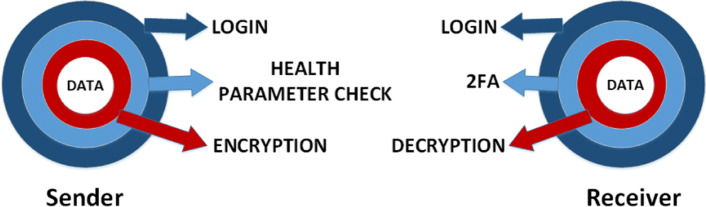
Zero trust principles in the proposed model

Zero trust principles ensure verification of users (and their devices) and data security (via encryption) in different layers as shown in Fig. 1.

The sender sends sensitive data to the intended receiver safely by going through the following three layers of security [32]:

- Login : This provides outermost layer of security which involves authentication of the sender using their login credentials like username and password via their end device, which in this case is the PC connected to the medical imaging device.
- Health Parameter Check : This is the second layer of security which involves checking the health parameter of the sender’s device before sending the data. This helps to detect whether the device is up to date on the latest security patches and precautions. This in turn ensures that the device has not been hacked or compromised.
- Encryption : This is the innermost layer of security which deals with data encryption. After the sender successfully passes the previous two layers, the data to be sent is encrypted with receiver’s public key- thus making it accessible only to the intended receiver possessing the key.

Again, the receiver will be able to access their data through a web page or app interface by undergoing the following security layers:

- Login : This provides the outermost layer of security which involves authentication of the receiver using their login credentials. However, this does not give them immediate access.
- 2FA : This is the second layer of security which involves undergoing two factor authentication (2FA) in order to enter the system. An authentication app will generate a code, which they must also input in order to gain access. Thus attackers cannot easily access a person’s device or online account because knowing the victim’s password alone is not enough. Two-factor authentication adds this additional layer of security to the authentication process [33].
- Decryption : This is the innermost layer of security which involves decryption of data. After completing the previous two steps,the receiver can view the encrypted data sent to them after decrypting it with their private key. Thus only the receiver can see their data.

### Blockchain

**Fig. 2:**
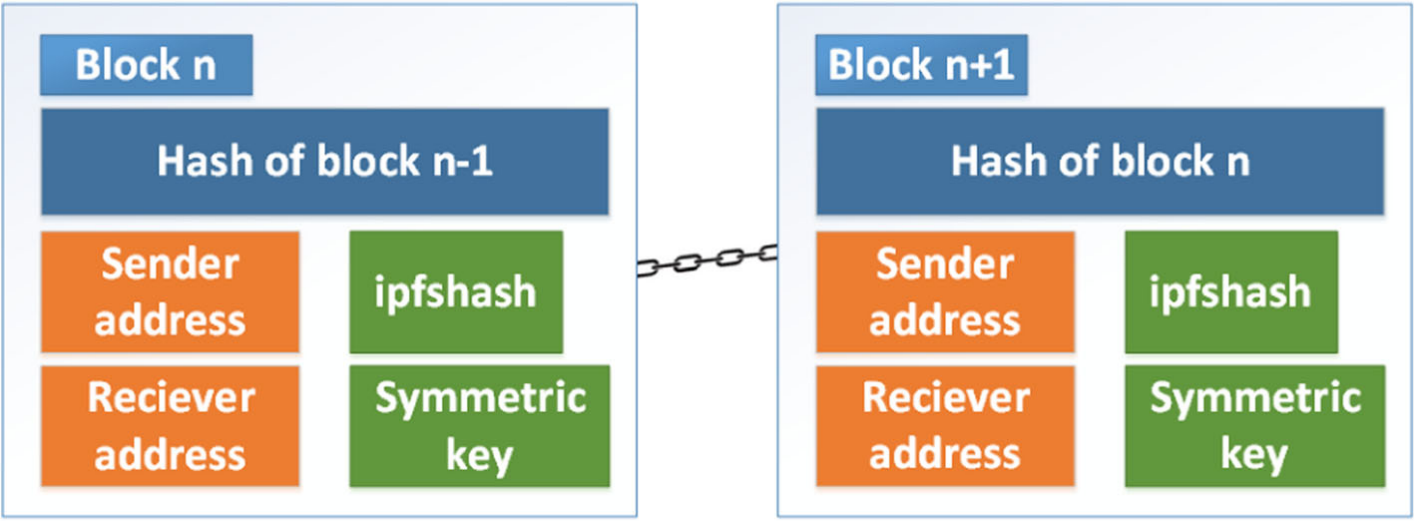
Block structure used in the proposed model

Blockchain is used in the model primarily to keep a record of every transaction taking place. The smart contracts in blockchain help to enable role-based access control which allows individuals to perform activities they are given permission for. To increase scalability, only hash of the data is stored in blockchain while the actual data is stored off chain in IPFS (a distributed file system and storage platform). It is decentralized so there is no single point of failure, and all trust is not put on a single node [34]. Moreover, it has been found to be the most suitable for blockchain than any other off chain storage (Swarm, StorJ, CoAP) [35]. Transaction will take place between the sender and the receiver of data. Each block consists of hash of the previous block, address of the sender, address of the receiver, symmetric key, ipfshash (hash generated by IPFS when image is uploaded) as shown in Fig. 2.

**Fig. 3:**
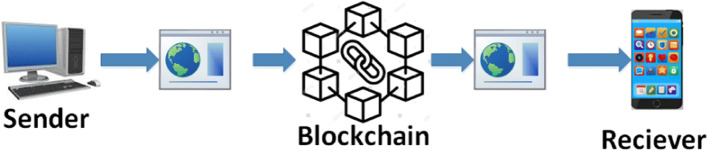
Blockchain usage in the proposed model

Whenever the sender requests to upload data, smart contract is called. This prompts the creation of a block, which is added to the blockchain only if the nodes participating in the blockchain network approve the addition of this node to the blockchain. If the block is successfully added then the receiver can retrieve their data. The sender and receiver will interact with the blockchain indirectly, through web pages. Since the primary focus of this research is to make the system as trustless and decentralised as possible, the proposed model will use a public blockchain like Ethereum which uses Proof of Work consensus mechanism to validate nodes. Figure 3 shows how blockchain interacts with the front end of the proposed model.

### Combination of blockchain and zero trust principles

**Fig. 4:**
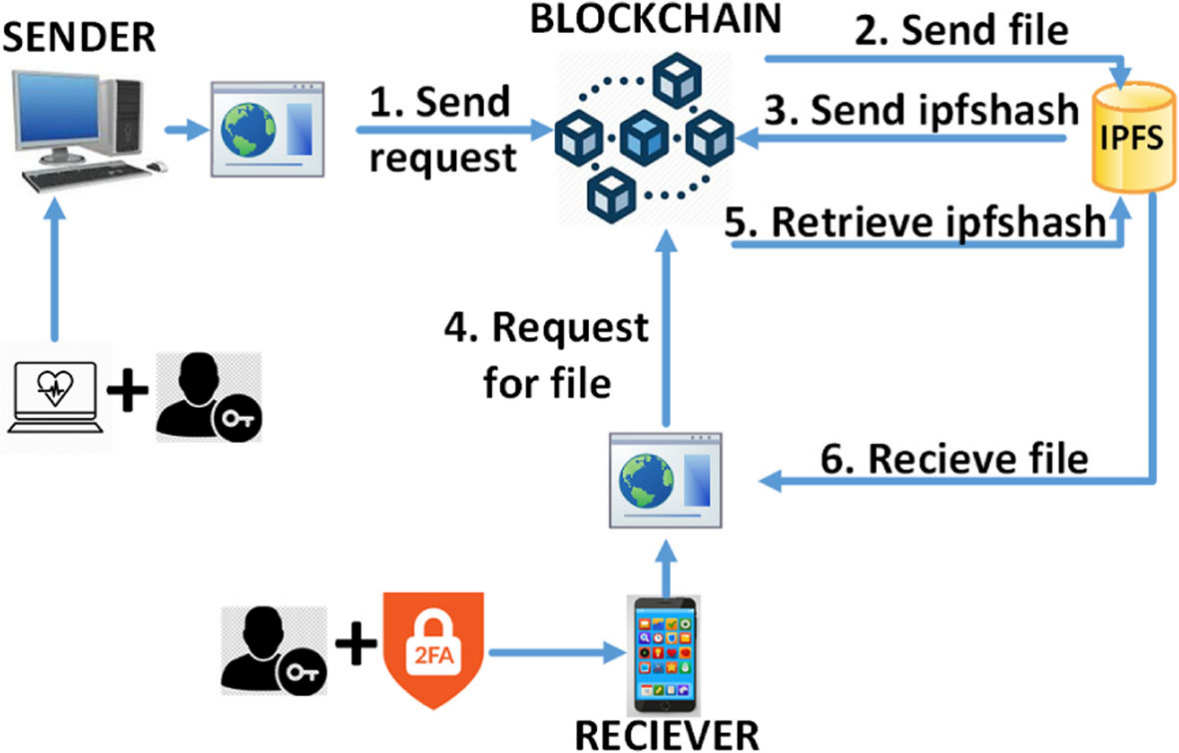
Integration of zero trust principles with blockchain in the proposed model
- Step 2 (Send file) : The file itself will be encrypted with a symmetric key and stored in IPFS. The corresponding hash of the file, referred to as ipfshash, is instantly generated. This hash denotes the location of the file in IPFS.
- Step 3 (Send ipfshash) : The generated ipfshash will be digitally signed with the sender’s private key and then encrypted using the receiver’s public key. A block will be created in the blockchain containing the elements mentioned in Fig. 2.
- Step 4 (Request for file) : After the receiver goes through the first two outer layers of security, Login and Two factor authentication (as shown in Fig. 1), the smart contract compares their authorization roles and privileges. If available, they will be able to request for file retrieval.
- Step 5 (Retrieve ipfshash) : If the request for file sent by the receiver contains the correct private key, the encrypted ipfshash retrieved from the blockchain will be decrypted and later verified with the sender’s public key.
- Step 6 (Receive file) : The encrypted file will be retrieved from IPFS with the help of the ipfshash and later decrypted with the symmetric key. Only then will the user be able to view the file they had requested for, in their end device.

The concepts of zero trust principles and blockchain are integrated to propose the model as shown in Fig. 4. Blockchain is used to ensure decentralization and immutability of data while zero trust principles are used for access control and authorization. Considering the cases of sending medical images, the functionalities of the proposed model are discussed below:

- Step 1 (Send request) : After the sender goes through the first two outer layers of security, Login and Health Parameters Checkup (as shown in Fig. 1), the smart contract checks their authorization roles and privileges. If available, the request is processed and they will be able to send a file.

The user can similarly share this file with other actors in the system, as a sender, if they have the permission to do so.

The system is dependent on the use of private and public key pairs for verification, validation, signing and cryptography. These can be assigned/generated using the user’s National Identity or Social Security Number with help from a Certification Authority - thus making them unique and legitimate [27].

## Results

The proposed system is developed, its security and performance evaluation and consequent implications are analysed.

### Implementation

**Fig. 5:**
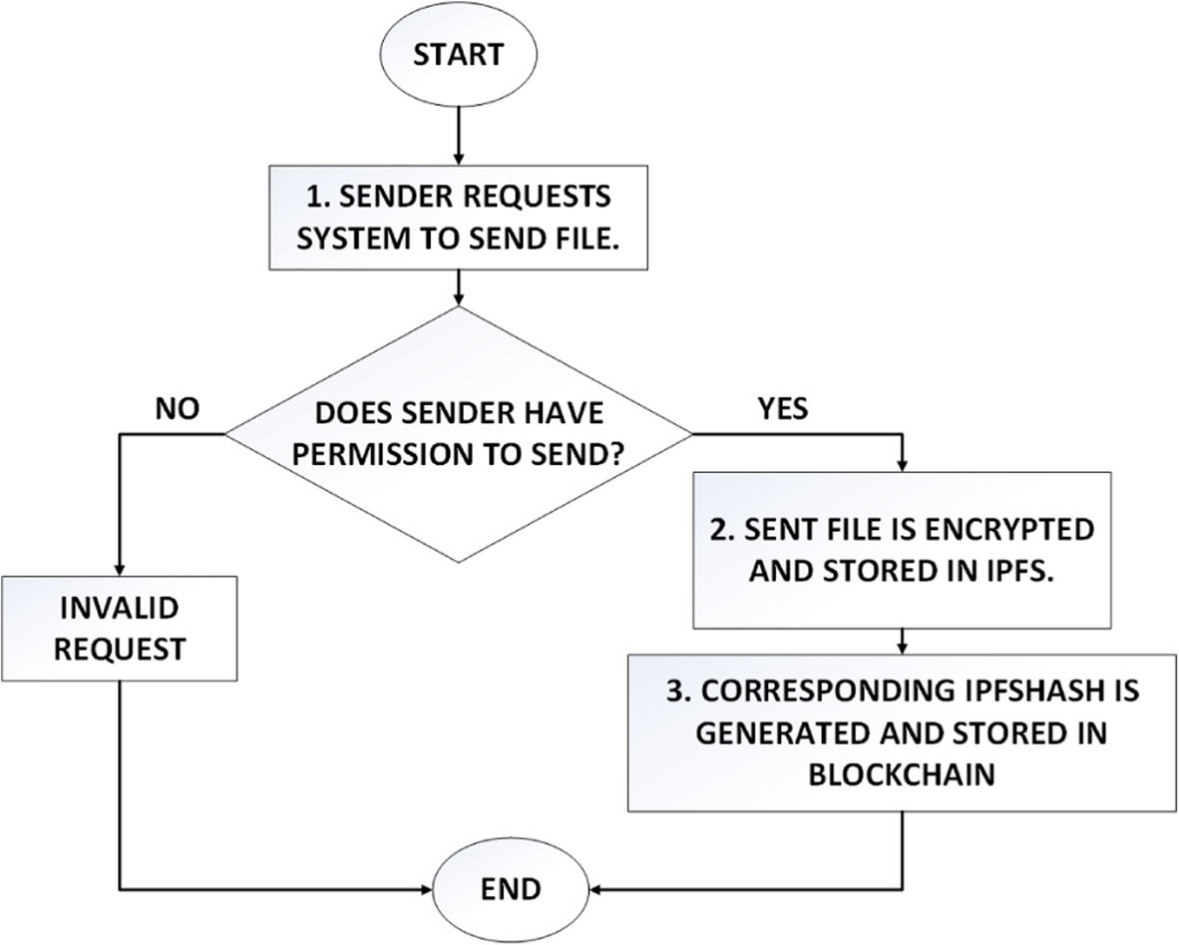
Workflow diagram shows the steps that take place when sender wants to send a file

**Fig. 6:**
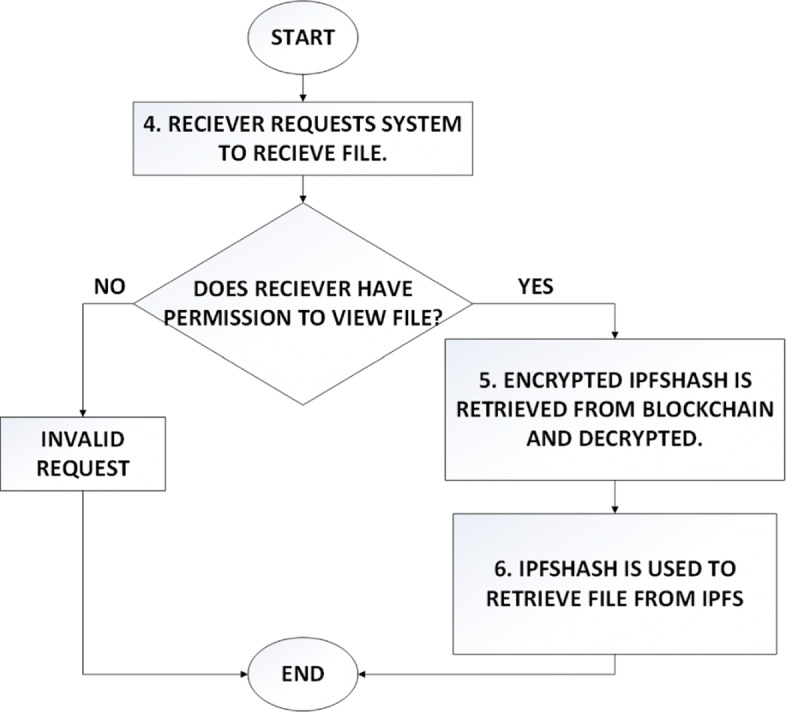
Workflow diagram shows the steps that take place when receiver wants to retrieve the file

In accordance with the proposed model, a decentralised web application was developed which allows a medical technologist to send medical image files to a patient. The file itself is stored off chain in IPFS while blocks in blockchain store the corresponding ipfshash. The workflow of the system from the perspective of sender and receiver is illustrated in Figs. 5 and 6.

A few tools were utilised to develop the model. Firstly, Ganache was used to run a local blockchain. It provides ten free accounts with fake Ether that were used to carry out the transactions. The private and public keys of these accounts were used for encryption and decryption. Secondly, Metamask was used to connect to the local Ethereum network. The system was connected to an IPFS node instance with the help of Infura for uploading image files. Thirdly, the client-side website was created to communicate with the smart contract with the help of Truffle framework. And finally, Solidity programming language was used to write the smart contract. Table 1 shows the specifications of the simulation platform.

**Table 1:** Simulation Platform

**System**	**Specification**
Operating System	Windows 10 Pro
Memory (RAM)	4.00 GB
Processor	Intel(R) Core(TM) i7-4600U CPU *@* 2.10 GHz

The smart contract contains a few role based functions. AddUser can only be called by the admin. This function is used to register the users (patient and medical technologist) and provide them with respective privileges (See Algorithm 1). Send function takes encrypted ipfshash and the recipient’s address as parameters and adds this new data to the sender’s image array (See Algorithm 2). And finally, the Get function takes an image array index as parameter and returns the corresponding encrypted ipfshash sent by the medical technologist (See Algorithm 3). The smart contract was tested and debugged using Truffle and Remix (a Solidity IDE used to write, compile and debug Solidity code).


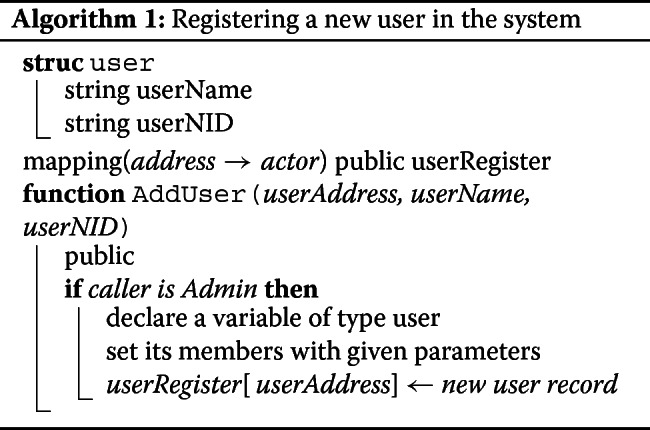



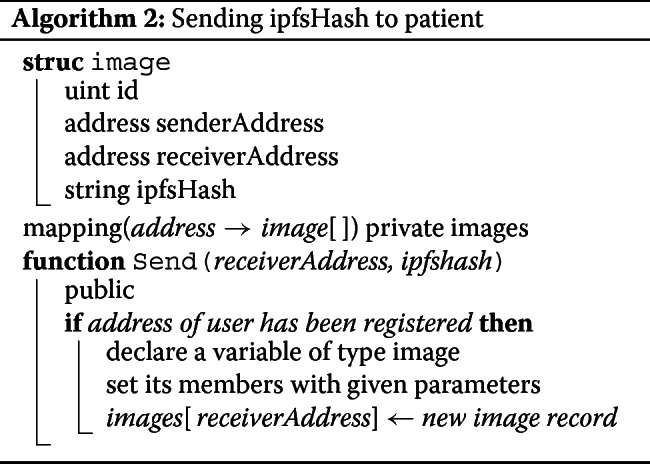



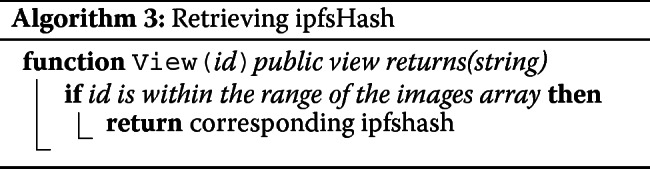


### Security analysis

The implemented model is analysed below to see how it can effectively meet with the aforementioned design goals:

*(a) Data security:* The security of web page login systems can be compromised by an active network attacker, as login credentials are shared over HTTP or third party resources [36]. To add more security and bypass this vulnerability, two factor authentication is used. According to Dmitrienko et al. [37], access to the OTP (One Time Password) is only possible if the interceptor has the user’s mobile device. As two factor authentication is the second step in accessing the web page after Login, it provides a barrier to potential threats by giving access upon receiving the OTP.

Before data can be sent, various health parameters of the device are checked. This ensures that the device that will send the data is not compromised in any way and is up to date on the latest security patches and precautions. Data is encrypted and sent to the IPFS only if all the device health requirements are met.

The system uses blockchain to keep an immutable audit trail of data access instances. Tsung-Ting et al. [38] states that the use of blockchain in medical systems protects data and is virtually unbreakable unless a 51 percent attack occurs.

Moreover, IPFS ensures secured data transfer among peers by providing secure filesharing and encrypted communication [39]. All the medical records are stored in IPFS after asymmetric encryption and data integrity is achieved by the digital signature of the sender.

*(b) Role-based access control:* In the proposed model, smart contract assigns users with different roles associated with different functions and privileges. It makes sure users cannot disguise their roles by acting as “autonomous agents” running exactly as programmed [40]. For example: only the Admin is allowed to add users in the system and assign them with different roles. Thus, users are only allowed to perform activities based on their role and can only access files that they own or have the permission to view. As a result, data ownership is also ensured.

*(c) Decentralization:* The data is decentralized both on and off chain, through blockchain and IPFS, both of which involve peer-to-peer verification and eliminate central control. According to Bashir et al. [41], decentralization is the distribution of control to end devices as opposed to a central authority. This removes single point of failure and more importantly, eliminates trust from central authority.

### Performance analysis

Medical records, especially medical imaging data, are relatively large in size. Storing them directly in blockchain is not feasible in terms of cost, space and time [42]. For this reason, the proposed system ensures scalability by storing the encrypted data off chain in IPFS and only the corresponding ipfshash in blockchain.

**Fig. 7:**
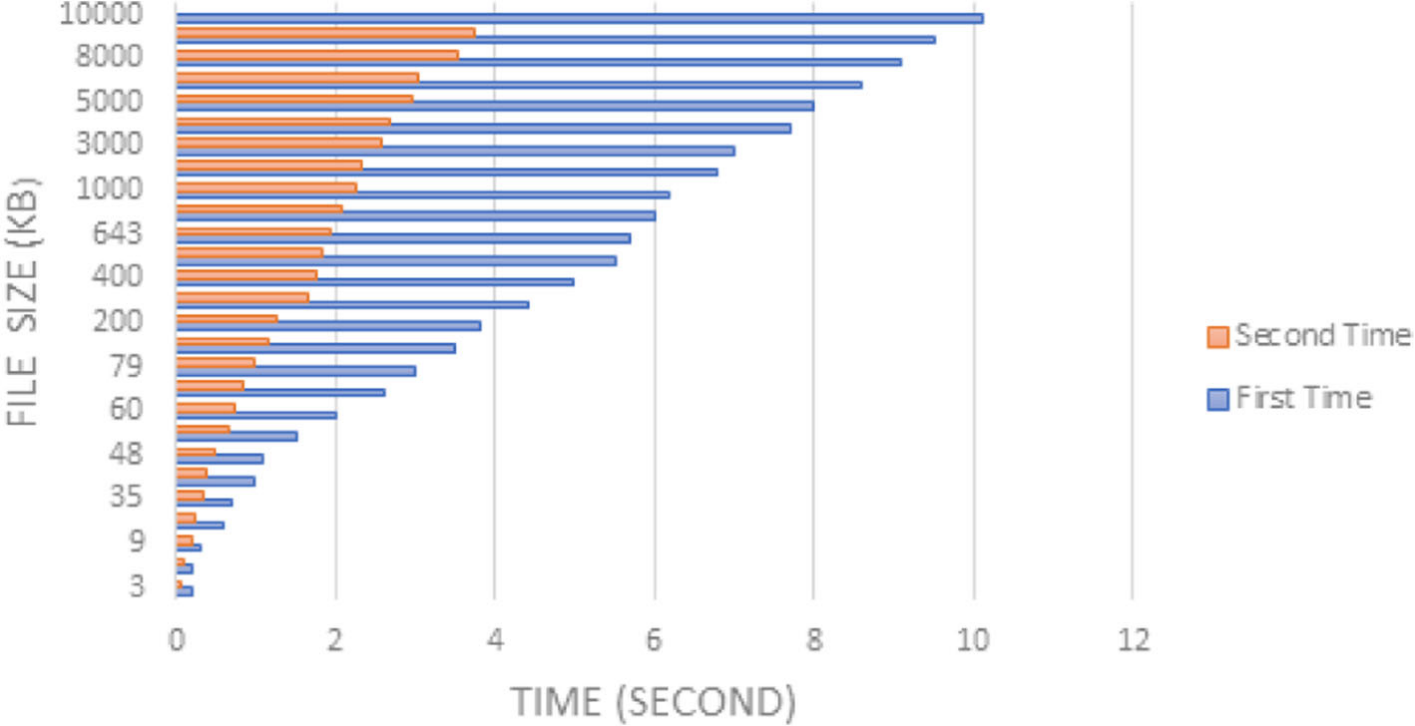
Difference in latency when same files are retrieved for the first and second time

In order to evaluate the system performance and efficiency, the time taken to retrieve the image files from IPFS was measured. The aforementioned data is presented in Table 2. Although larger files start to load at around 5 to 7 seconds, the full image can sometimes take as long as 1 minute to show up on screen. The gathered data has been illustrated in the form of a chart in Fig. 7. From the chart, it is evident that file size and latency are directly proportional, the bigger the file, the longer it takes to retrieve. However, it is also seen that, retrieval of the same files from IPFS the second time is significantly a lot faster because IPFS caches the data locally after the first delivery, thus, reducing the latency. Either way, improvements need to be made to retrieve larger files quicker.

**Table 2:** Retrieval time of varying file sizes

**Size (kB)**	**Type**	**Mean Retrieval Time (s)**	**Mean Retrieval Time for 2 **^***nd***^** Request (s)**
≤60	Small	0.75	0.31
61 - 250	Medium	3.54	1.02
251 - 10000	Large	6.83	2.42

## Discussion

This paper provides a brief overview of the proposed decentralised trustless model which aims to tackle a lot of the security issues related to sharing and storing of medical records and images in an electronic health system. This has been done by the incorporation of blockchain and zero trust principles. The model was simulated by deploying a decentralized web application which helps to share and store medical records and images between users. The proposed model was fully decentralised and scalable. It improves data security by ensuring role-based access and encryption.

This research, however, has a few limitations. One of the drawbacks of the proposed model is the network speed. Since each transaction requires peer-to-peer verification, it becomes time-consuming especially in a public blockchain with many nodes. And although Proof of Work ensures total decentralisation, it has a high demand on node performance and wastes energy. Apart from that, key management can become a bit cumbersome for the users especially during the loss of a key.

## Conclusions

The aim of this study was to enhance the security of medical records and images (before, during and after) transmission through a combination of blockchain and zero trust principles. Blockchain was used to keep an audit trail of medical/health data transmissions for future examination. Zero trust principles were employed in keeping medical data safe during transmission and enhancing security on the user’s side.

In the future, the plan involves implementation of the total framework and its deployment on the Ethereum blockchain to test out its scalability and efficiency in the real world. The plan also includes incorporation of all the proposed security layers, testing and analysing their effectiveness quantitatively by deploying it in an actual industry. One of the future objectives also involves finding ways to make the system a lot faster and user friendly.

## Data Availability

The data set used and/or analysed during the current study are available from the corresponding author on reasonable request.

## Abbreviations

IPFS: Inter Planetary File System
MRI: Magnetic’Resonance Imaging
CT: Computerized Tomography
e-health: Electronic Health
2FA: Two Factor Authentication
EMR: Electronic Medical Record
PBFT: Practical Byzantine FaultTolerance
IoT: Internet of Things
ipfshash: Hash generated by IPFS when image is uploaded
